# The quality of systematic reviews of health-related outcome measurement instruments

**DOI:** 10.1007/s11136-015-1122-4

**Published:** 2015-09-07

**Authors:** C. B. Terwee, C. A. C. Prinsen, M. G. Ricci Garotti, A. Suman, H. C. W. de Vet, L. B. Mokkink

**Affiliations:** Department of Epidemiology and Biostatistics and the EMGO+ Institute for Health and Care Research, VU University Medical Center, P.O. Box 7057, 1007 MB Amsterdam, The Netherlands; Department of Psychology, University of Bologna, Bologna, Italy; Department of Public Health and the EMGO+ Institute for Health and Care Research, VU University Medical Center, Amsterdam, The Netherlands

**Keywords:** Systematic review, Outcome measurement instruments, Measurement properties, Reliability, Validity

## Abstract

**Background:**

Systematic reviews of outcome measurement instruments are important tools for the selection of instruments for research and clinical practice. Our aim was to assess the quality of systematic reviews of health-related outcome measurement instruments and to determine whether the quality has improved since our previous study in 2007.

**Methods:**

A systematic literature search was performed in MEDLINE and EMBASE between July 1, 2013, and June 19, 2014. The quality of the reviews was rated using a study-specific checklist.

**Results:**

A total of 102 reviews were included. In many reviews the search strategy was considered not comprehensive; in only 59 % of the reviews a search was performed in EMBASE and in about half of the reviews there was doubt about the comprehensiveness of the search terms used for type of measurement instruments and measurement properties. In 41 % of the reviews, compared to 30 % in our previous study, the methodological quality of the included studies was assessed. In 58 %, compared to 55 %, the quality of the included instruments was assessed. In 42 %, compared to 7 %, a data synthesis was performed in which the results from multiple studies on the same instrument were somehow combined.

**Conclusion:**

Despite a clear improvement in the quality of systematic reviews of outcome measurement instruments in comparison with our previous study in 2007, there is still room for improvement with regard to the search strategy, and especially the quality assessment of the included studies and the included instruments, and the data synthesis.

## Introduction

Health-related outcome measurement instruments are used to evaluate the effects of disease and treatment over time. Systematic reviews of health-related outcome measurement instruments are important tools for the selection of instruments for research and clinical practice and for identifying gaps in knowledge on the quality of outcome measurement instruments, i.e., their measurement properties [1]. Systematic reviews of outcome measurement instruments are being used for a number of purposes: (1) for selecting outcome measurement instruments for monitoring patients in clinical practice; (2) for selecting outcome measurement instruments in the design of new research projects; (3) as a source for evidence on the measurement properties of the outcome measurement instruments used in clinical trials and other (submitted) studies; and (4) for selecting outcome measurement instruments for outcomes included in Core Outcome Sets (COS, i.e., an agreed set of outcomes that should be measured and reported in all clinical trials of a specific condition [2]).

Systematic reviews should be of high methodological quality to provide a comprehensive and unbiased overview of the measurement properties of the available outcome measurement instruments. In general, a high-quality systematic review consists of a comprehensive search strategy in multiple databases, a selection of abstracts and full-text articles by at least two independent reviewers, a methodological quality assessment of the included studies, and a systematic evaluation and interpretation of the results of the included studies (www.cochrane-handbook.org).

In 2007, we assessed the methodological quality of 148 systematic reviews of health-related outcome measurement instruments published up to March 2007 [1]. Three major limitations were identified. First, the search strategy was often of low quality: in 22 % of the reviews a search was performed in only one database, the search strategy was often poorly described, and in more than 70 % of the reviews it was not reported whether the article selection and data extraction was done by two independent reviewers. Second, in only 30 % of the reviews the methodological quality of the included studies on measurement properties was (partly) evaluated. Third, in only 55 % of the reviews (some) criteria were used to evaluate the quality of the included instruments.

Since this study, the COnsensus-based Standards for the selection of health Measurement INstruments (COSMIN) initiative developed tools to improve the quality of systematic reviews of outcome measurement instruments. COSMIN is an international group of researchers that aim to improve the selection of health measurement instruments for research and clinical practice. By means of an international Delphi study, COSMIN developed consensus-based standards for assessing the methodological quality of studies on measurement properties, including design requirements and preferred statistical methods [3]. The standards were operationalized into a user-friendly checklist that can be used in systematic reviews of outcome measurement instruments to evaluate the quality of the included studies on measurement properties [4]. The COSMIN checklist was published in 2010 and has been used in more than 60 systematic reviews of outcome measurement instruments since then. In addition, COSMIN researchers developed a protocol for systematic reviews of outcome measurement instruments, made available through the COSMIN website (www.cosmin.nl).

The aim of this study was to assess the current state of the quality of systematic reviews of health-related outcome measurement instruments and to determine whether the methodological quality of these reviews has been improved over time.

## Methods

A systematic literature search was performed on June 19, 2014, in MEDLINE (using PubMed) and EMBASE (using www.embase.com) to identify all systematic reviews of health-related outcome measurement instruments published between July 1, 2013, and June 19, 2014. We aimed to identify about 100 reviews to make a comparison with the reviews from our study in 2007. We included reviews published from 2013 onwards that had the potential to have incorporated the COSMIN checklist, which was published in 2010 [3].

The search strategy consisted of search terms for systematic reviews, search terms for measurement instruments, and a validated methodological search filter for measurement properties [5]. References of included reviews were checked for additional relevant reviews. The full search strategy is provided in ‘Appendix.’

The following inclusion criteria were used: (1) the study should be a systematic review (we considered a review to be systematic if at least one search in an electronic database was performed); (2) the aim of the review should be to identify all outcome measurement instruments of interest and to summarize the evidence on their measurement properties; (3) the construct of interest of the review should be (aspects of) health status, defined as (a) biological and physiological processes, OR (b) symptoms, OR (c) physical functioning, OR (d) social/psychological functioning, OR (e) general health perceptions, OR (f) health-related quality of life (based on the model of Wilson & Cleary [6] ); (4) the study population should contain humans (patients or general population); (5) the instruments of interest should be outcome measurement instruments, defined as instruments which can be/are applied in longitudinal studies to monitor changes in health over time (the outcome measure is the dependent variable); and (6) the study should evaluate and report on at least one or more measurement properties of the included instruments.

The following exclusion criteria were used: (1) reviews of diagnostic or screening instruments which are not used to evaluate the effects of disease and treatment over time; (2) prognostic reviews, i.e., reviews of prognostic studies (prediction models) which aim to predict an outcome using multi-variable analysis; (3) non-English articles; and (4) reviews of only one, or only the most commonly used measurement instruments, or reviews that only included randomized controlled trials (RCTs).

Titles and abstracts were screened by two reviewers independently (CT and MR or CP and MR), and consensus was reached. Full-text articles were screened by two reviewers independently (different couples of CT, CP, MR, and LM), and consensus was reached by discussion among the two reviewers.

A study-specific checklist was developed for data extraction and to evaluate the quality of the systematic reviews of health-related outcome measurement instruments, based on criteria used in our previous study [1], existing guidelines for the appraisal of systematic reviews of clinical trials (Cochrane handbook (http://www.cochrane-handbook.org) [7] and diagnostic studies (Cochrane handbook (http://srdta.cochrane.org/handbook-dta-reviews), and a checklist for assessing the methodological quality of systematic reviews (AMSTAR) [8]). Our checklist contains items on the quality of the research question (inclusion of the construct, target population, measurement instruments and measurement properties of interest), the search strategy (number and kind of databases searched, use of a time window, use of search terms for measurement properties and measurement instruments, language limitations and reference checking), whether the inclusion and exclusion criteria were clearly described, whether and how quality assessment of the included studies was performed, whether and how quality assessment of the included outcome measurement instruments was performed, whether and how data synthesis was performed, whether article selection, data extraction, and quality assessment was done by two reviewers independently, whether evidence-based recommendations were provided, and whether conflict of interest statements were included (Table 1). The quality assessment criteria were similar to those used in our study in 2007. However, in the current study we used additional criteria that were not assessed in our study in 2007. This is indicated in Table 1.

**Table 1:** Quality assessment of systematic reviews of outcome measurement instruments

Quality aspects	% current study	% study of 2007 [1]^a^
Elements included in the research aim		
Construct of interest	94	
Population of interest	88	
Type of measurement instrument of interest	52	
Measurement properties of interest	81	
All available instruments included	52	
Only instruments included that have at least some evidence of measurement properties	48	
Search strategy described	93	84
No search terms or validated search filter used for		
Measurement properties	64	
Type of instrument	25	
Number of databases searched [median (range)]	4 (1–15)	
Search in at least 2 databases	92	76
MEDLINE/PubMed	92	93
EMBASE	59	35
Additional databases	87	57
Reference checking used	65	
No time limits used or good arguments for a time limit	72	
No language restrictions used	26	79
Inclusion and exclusion criteria clearly described	86	72
Reasons for excluding articles reported	55	
Abstract selection by at least 2 reviewers?		
Yes	41	
No	21	
Unclear	38	
Full-text article selection by at least 2 reviewers?		
Yes	38	
No	13	
Unclear	48	
Abstract and full-text article selection by at least 2 reviewers?		
Yes	29	22
No	12	3
Unclear	59	75
Methodological quality of studies assessed	41	30
Quality assessment of studies done by at least 2 reviewers		
Yes	60	
No	12	
Unclear	28	
Data on measurement properties extracted by at least 2 reviewers		
Yes	25	25
No	13	4
Unclear	62	71
Quality of the instrument (measurement properties) assessed	58	55
Quality assessment of the instrument by at least 2 reviewers		
Yes	33	
No	5	
Unclear	62	
Results from multiple studies on the same instrument somehow combined (e.g., best evidence synthesis or pooling)		
Yes, clearly described	20	7^b^
Yes, but unclear how	22	
No	58	
Data synthesis was performed…		
Per measurement property	79	
Only for domains (reliability, validity, responsiveness)	9	
Only for the whole instrument	12	
Recommendation provided for the best instrument		
One instrument is recommended per construct	23	
More instruments are recommended per construct	26	
No recommendation for the best instrument	51	
Results for the measurement properties reported as raw data		
Yes	56	
Partly	13	
No	31	
Number of measurement properties reported [median (range)]	6 (1–9)	
Conflict of interest or funding source declared	81	
One of the authors of the review is also the developer of one of the instruments evaluated in the review	9	

^a^Not all items were evaluated in the study in 2007

^b^Yes (clearly described or unclear how combined)

We also counted the number of measurement properties reported in the reviews, using the COSMIN taxonomy (nine measurement properties) [9]. The data extraction and quality appraisal was done by two reviewers independently (different couples of CT, CP, MR, and LM), and consensus was reached by discussion among the two reviewers. If consensus was not reached, a third reviewer was included and the final decision was made based on consensus among the three reviewers.

The results of the study were compared to the results of our previous study, which used a similar search strategy (although not exactly the same search terms for measurement properties as the search filter for measurement properties did not exist yet), the same inclusion criteria, and included all systematic reviews of health-related outcome measurement instruments (*n* = 148) published up to March 2007 [1].

## Results

**Fig. 1:**
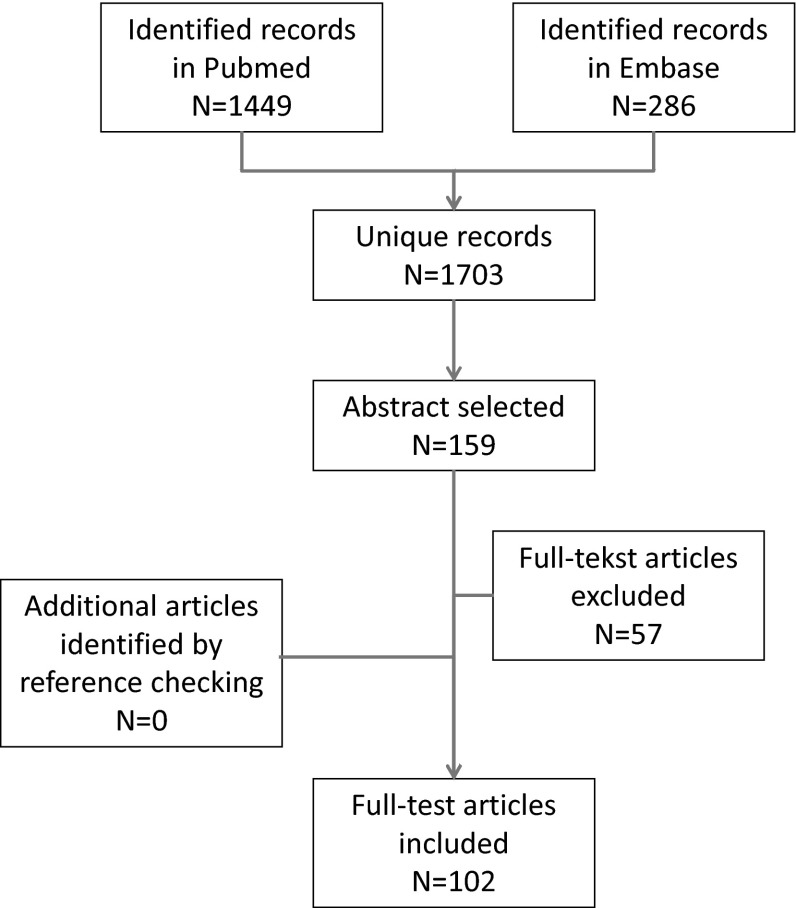
Flow chart of abstract and article selection

The search strategy yielded 1703 unique records, of which 157 abstracts were selected for retrieving the full-text articles. From these 157 articles, 55 articles were excluded because they were about non-health-related constructs or did not provide information on the measurement properties. The remaining 102 systematic reviews of outcome measurement instruments were included [10–59, 60–109, 110, 111]. A flow chart of the abstract and article selection process is provided in Fig. 1.

The results of the quality appraisal of the systematic reviews of outcome measurement instruments are presented in Table 1 and compared with the results of our previous study for items for which this was possible. The construct, target population, and measurement properties of interest of the review were clearly described in the research aim in more than 80 % of the reviews. However, in only 52 % of the reviews the type of measurement instruments of interest was described in the research aim.

The search strategy was described at least to some extent in 93 % of the reviews, as compared to 84 % in 2007. In 54 % of the reviews, no search terms for measurement properties were used. In 25 % of the reviews no search terms for type of measurement instruments of interest were used.

The median number of databases searched was four (range 1–15), and in 92 % of the reviews a search in at least two databases was performed. This percentage was 76 % in 2007. MEDLINE or PubMed was used in 92 % of the reviews (93 % in 2007), and EMBASE was used in 59 % of the reviews (35 % in 2007).

The selection of abstracts and full-text articles was performed by at least two reviewers independently in 29 % of the reviews, as compared to 22 % in 2007. In 59 % of the reviews (75 % in 2007), it was unclear or not described.

In 41 % of the reviews, the methodological quality of the included studies was assessed, as compared to 30 % in 2007. In 60 % (*n* = 25) of these reviews [10, 18, 21, 26, 29–31, 39, 50, 52, 54, 57, 63, 64, 68, 78, 84, 87, 91, 96, 102, 104, 107, 108, 110] the COSMIN checklist [3, 4] was used. In 60 % (*n* = 25) of the reviews that assessed the quality of the studies, the quality assessment was performed by at least two reviewers independently.

In 58 % of the reviews the quality of the included outcome measurement instruments (i.e., their measurement properties) was assessed, as compared to 55 % in 2007. In 36 % (*n* = 21) of these reviews [10, 15, 18, 26, 29, 35, 39, 50, 54, 57, 62, 63, 68, 78, 84, 85, 91, 93, 97, 100, 108], quality criteria published by Terwee et al. [112] were used. In 32 % (*n* = 19) of these reviews, the quality assessment was performed by at least two reviewers independently.

In 42 % of the reviews some kind of data synthesis was performed in which the results from multiple studies on the same instrument were combined according to predefined criteria, as compared to 7 % in 2007. However, in only about half of these reviews (*n* = 20) [10, 12, 13, 29, 39, 50, 54, 63, 68, 70, 77, 78, 81, 82, 84, 95, 102, 104, 108, 109], it was clearly described how this was done. In 81 % (*n* = 34) of the reviews that performed data synthesis, the data synthesis was performed per measurement property separately. In 13 reviews [10, 13, 29, 39, 50, 54, 57, 63, 68, 78, 91, 104, 108], a best evidence syntheses was used based on methods used in Cochrane reviews of clinical trials, in which the number, methodological quality, and consistency of the results of the included studies are taken into account.

In 49 % of the reviews clear recommendations were provided for either one or multiple outcome measurement instruments per construct that were considered the best. In about half of these reviews (*n* = 23) [14, 16, 24, 29, 30, 37, 40, 42, 45, 50, 52–54, 63, 69, 72, 87, 90, 96, 101, 102, 110, 111] a recommendation was provided for one best outcome measurement instrument per construct of interest.

In 81 % of the reviews a conflict of interest statement was provided. In nine of these reviews [31, 36, 48, 51, 53, 59, 76, 79, 93] (one of) the authors of the review was involved in the development of one of the instruments included in the review; in only one review [31] this was explicitly stated and the instrument for which this was the case was rated by an independent reviewer. In four reviews [36, 48, 59, 79] the instrument that was recommended as (one of) the best instruments was developed by (one of) the authors of the review, but the involvement of the authors in the development of the instrument was not mentioned in the conflict of interest statement.

## Discussion

Despite some clear improvements in the quality of systematic reviews of outcome measurement instruments since our study in 2007 [1], there is still room for improvement with regard to the search strategy, and especially with regard to the quality assessment of the included studies, the quality assessment of the included instruments, and the data synthesis.

In many cases the search strategy was likely to be incomprehensive, for several reasons: First, in only 59 % of the reviews a search was performed in EMBASE (35 % in 2007 [1]). In several systematic reviews of outcome measurement instruments that we performed, we found two or three relevant articles in EMBASE that were not found in MEDLINE [113–115]. Therefore we recommend reviewers to always search at least MEDLINE and EMBASE. Second, in only 25 % of the reviews, no search terms for type of measurement instruments were used. It is understandable that many reviews use search terms for measurement instruments because without search terms for type of measurement instruments, often too many abstracts need to be screened. However, in about half of the reviews we had doubts about the comprehensiveness of the search terms used for type of measurement instruments. For example, a review on questionnaires for assessment of gastroesophageal reflux disease used the search terms: (‘Questionnaires’[Mesh] OR questionnaire*[ti] OR scale*[ti]) [16] for type of instruments. We consider it doubtful whether all questionnaires will be found with these terms, because authors may use other terms like ‘instrument,’ ‘outcome measure’ or ‘patient-reported outcome.’ In general it is preferred to use no search terms for measurement instruments, to avoid missing studies. However, if this leads to too many search results, a comprehensive block of search terms need to be developed. For reviews on patient-reported outcome measures, a search filter developed by a group from Oxford University (available from www.cosmin.nl) could be used. Third, in 28 % of the reviews we had doubts about the comprehensiveness of the search terms used for measurement properties. For example, a review on outcome measurement instruments for upper limb function in multiple sclerosis used the search terms (psychometric properties OR psychometrics OR validity OR reliability OR test–retest OR responsiveness) [51] for measurement properties. We consider it doubtful whether all studies on measurement properties will be found with these terms, because authors may use other terms like ‘measurement properties,’ ‘clinimetric properties,’ or sensitivity to change.’ There is large variation in terminology used for measurement properties, and studies on measurement properties are poorly indexed in databases like MEDLINE [5]. Again, it is preferred to use no search terms for measurement properties to avoid missing studies. However, if this is not feasible, a highly sensitive search filter for finding studies on measurement properties could be used, which was published in 2009 [5] (also available from www.cosmin.nl). This filter was used in only 10 reviews. Fifty-five reviews used no search terms for measurement properties, which is considered to be a comprehensive strategy because all studies will be screened, but time consuming. Using the search filter for finding studies on measurement properties reduces the number needed to read with about 75 % and has a high sensitivity of about 95–97 % [5]. Fourth, we could not rate the comprehensiveness of the search terms for the construct and target population of interest, because this requires expertise and knowledge of the construct and target population of interest. In many cases it is useful to consult a clinical librarian, which was not reported in most of these reviews. A recent study showed that librarian and information specialist authorship was associated with better reported systematic review search quality [116]. Finally, in 36 % of the reviews reference checking was not performed. It is generally recommended for systematic reviews to check references of included articles. If several relevant articles are found with reference checking, the search strategy was likely incomprehensive and should be adapted.

There is important room for improvement with regard to the quality assessment of the included studies, which was performed in only 41 % of the reviews. This percentage was 30 % in our previous study [1], so it has improved, but not satisfactory. If the methodological quality of a study on the measurement properties of an instrument is inadequate, the results may be biased and the quality of the instrument may be underestimated or overestimated. The COSMIN checklist was used in 25 reviews. An additional 17 reviews used other checklists or recommendations such as QUADAS or ad hoc developed standards. Many of these standards seemed incomplete, for example do not include standards for all measurement properties, or were unclearly described. We recommend to use the COSMIN checklist because it is the only consensus-based checklist containing detailed standards for the preferred design characteristics and statistical methods of studies on measurement properties and includes a standardized rating system for scoring the quality of studies on measurement properties [3, 4, 117]. QUADAS was developed for rating the quality of studies on diagnostic measurement instruments [118, 119], not outcome measurement instruments, so it is less applicable for these kind of reviews.

There is also room for improvement in the quality assessment of the included outcome measurement instruments, which was performed in only 58 % of the reviews. This was 55 % in our previous study, so has not improved much. There is wide variation in how the quality of the instruments was assessed and which criteria for what constitutes good measurement properties were used. The most often used quality criteria were those published by Terwee et al. [112], which were used in 21 reviews. These criteria were not developed using a consensus procedure, but recently, international consensus was reached on these criteria (with minor modifications) in a collaborative study of the COSMIN and the Core Outcome Measures in Effectiveness Trials (COMET) initiative regarding the development of a guideline for selecting outcome measurement instruments for Core Outcome Sets [120].

In less than half of the reviews (42 %) a data synthesis was performed in which the results from multiple studies on the same instrument were somehow combined. In our previous study this was only 7 %. Data synthesis is an important step in a systematic review to develop evidence-based and transparent recommendations for the best instrument for a given context of use. The methodology of data synthesis of studies on measurement properties is not yet as thoroughly developed as it is for reviews of clinical trials, where GRADE recommendations are being used [121–123]. The data synthesis of studies on measurement properties is complex because it is different for each measurement property. For example, to rate the evidence for internal consistency, methods and results of factor analyses (dimensionality) as well as methods and results of internal consistency analyses (Cronbach’s alpha) should be considered and combined and this information may come from different studies. To rate the evidence for reliability, statistical pooling of intraclass correlation coefficients might be considered. It is not yet clear how the results of different construct validity or responsiveness studies can be combined, taking into account the strength and the number of hypotheses tested, the constructs being measured with the comparison instruments used, the quality of the comparison instruments, and the kind of subgroups being compared. It remains to be examined if the GRADE recommendations can also be applied, or perhaps in modified form, in systematic reviews of outcome measurement instruments.

Although it is not easy to perform a data synthesis, a review requires a transparent conclusion. Only 49 % of the reviews provided recommendations for the use of one or multiple outcome measurement instruments. We think it is important that reviews provide clear recommendations for the use of instruments because researchers and clinicians need to choose an instrument for their study or use in clinical practice, even when the information on certain measurement properties is scarce or lacking. A recommendation for the use of one instrument per construct and population of interest will facilitate uniformity in outcome reporting and, as a consequence, meta-analyses of studies. It is also important to discourage the use of instruments with evidence for poor measurement properties and to indicate which instruments need further research on their measurement properties.

Finally, it is important that reviewers clearly indicate their involvement in the development or validation of one of more of the included instruments in the review in a conflict of interest statement because this may have influenced their ratings of the included instruments and their recommendations.

Some limitations of this review should be acknowledged. First, no validated search filter was used for finding systematic reviews, such as the recommended health-evidence.ca systematic review search filter [124], because this filter includes the terms ‘meta-analysis’ and ‘intervention’, which were not relevant for our review. Our search terms were, however, quite similar to the remaining search terms of this filter, such as ‘systematic review.tw,’ so we believe our search was sufficiently sensitive. Second, in our previous review we also searched in PsycINFO but this yielded only 3 of the 148 included reviews. In this study we therefore decided to only use MEDLINE and EMBASE. Our aim was not to find all available systematic reviews but to compare the quality of a set of the most recently published reviews with a set of reviews published six or more years ago. Third, as in many of the included articles in our review, our search terms for type of measurement instruments may also not have been comprehensive, because many different terms are being used in the literature for measurement instruments. Ideally, no search terms should be used for type of measurement instruments but in our review that would have increased the number of records found in PubMed alone to more 160.000. Fourth, the quality assessment of the included reviews was hampered by poor reporting of methods used in the review, especially whether the abstracts and articles were selected by two independent reviewers, and how the data synthesis was performed. We had no time to contact the authors of the reviews for more information. Therefore, we may have underestimated the quality of some reviews.

We conclude that despite a clear improvement in the quality of systematic reviews of outcome measurement instruments in comparison with our previous study in 2007, there is still room for improvement regarding the search strategy, and especially with regard to the quality assessment of the included studies, the quality assessment of the included instruments, and the data synthesis. We recommend reviewers to use the tools developed by the COSMIN group, such as the search filter for finding studies on measurement properties and the COSMIN checklist for assessing the quality of the included studies. A protocol for performing systematic reviews of outcome measurement instruments is available from the authors (CT). The methodology of systematic reviews of outcome measurement instruments need to be further developed. The COSMIN group is currently working on a guideline for systematic reviews of outcome measurement instruments (an update of the currently available protocol). There is also room for improvement with regard to the reporting of systematic reviews of outcome measurement instruments. The development of guidelines for reporting systematic reviews of outcome measurement instruments merits attention in future research.

## Appendix: Search strategy

### Pubmed search June 19, 2014

#1: (instruments[tiab] OR scales[tiab] OR Questionnaires[tiab] OR measures[ti] OR methods[ti] OR outcome measurements[tiab] OR (tests[tiab] AND review[tiab]) OR Questionnaires[MeSH] OR interview[MeSH]).

#2 (systematic[sb] OR (literature AND search*) OR (Medline AND search*) OR review[ti]).

#3 (instrumentation[sh] OR methods[sh] OR “Validation Studies”[pt] OR “Comparative Study”[pt] OR “psychometrics”[MeSH] OR psychometr*[tiab] OR clinimetr*[tw] OR clinometr*[tw] OR “outcome assessment (health care)”[MeSH] OR “outcome assessment”[tiab] OR “outcome measure*”[tw] OR “observer variation”[MeSH] OR “observer variation”[tiab] OR “Health Status Indicators”[Mesh] OR “reproducibility of results”[MeSH] OR reproducib*[tiab] OR “discriminant analysis”[MeSH] OR reliab*[tiab] OR unreliab*[tiab] OR valid*[tiab] OR “coefficient of variation”[tiab] OR coefficient[tiab] OR homogeneity[tiab] OR homogeneous[tiab] OR “internal consistency”[tiab] OR (cronbach*[tiab] AND (alpha[tiab] OR alphas[tiab])) OR (item[tiab] AND (correlation*[tiab] OR selection*[tiab] OR reduction*[tiab])) OR agreement[tw] OR precision[tw] OR imprecision[tw] OR “precise values”[tw] OR test–retest[tiab] OR (test[tiab] AND retest[tiab]) OR (reliab*[tiab] AND (test[tiab] OR retest[tiab])) OR stability[tiab] OR interrater[tiab] OR inter-rater[tiab] OR intrarater[tiab] OR intra-rater[tiab] OR intertester[tiab] OR inter-tester[tiab] OR intratester[tiab] OR intra-tester[tiab] OR interobserver[tiab] OR inter-observer[tiab] OR intraobserver[tiab] OR intra-observer[tiab] OR intertechnician[tiab] OR inter-technician[tiab] OR intratechnician[tiab] OR intra-technician[tiab] OR interexaminer[tiab] OR inter-examiner[tiab] OR intraexaminer[tiab] OR intra-examiner[tiab] OR interassay[tiab] OR inter-assay[tiab] OR intraassay[tiab] OR intra-assay[tiab] OR interindividual[tiab] OR inter-individual[tiab] OR intraindividual[tiab] OR intra-individual[tiab] OR interparticipant[tiab] OR inter-participant[tiab] OR intraparticipant[tiab] OR intra-participant[tiab] OR kappa[tiab] OR kappa’s[tiab] OR kappas[tiab] OR repeatab*[tw] OR ((replicab*[tw] OR repeated[tw]) AND (measure[tw] OR measures[tw] OR findings[tw] OR result[tw] OR results[tw] OR test[tw] OR tests[tw])) OR generaliza*[tiab] OR generalisa*[tiab] OR concordance[tiab] OR (intraclass[tiab] AND correlation*[tiab]) OR discriminative[tiab] OR “known group”[tiab] OR “factor analysis”[tiab] OR “factor analyses”[tiab] OR “factor structure”[tiab] OR “factor structures”[tiab] OR dimension*[tiab] OR subscale*[tiab] OR (multitrait[tiab] AND scaling[tiab] AND (analysis[tiab] OR analyses[tiab])) OR “item discriminant”[tiab] OR “interscale correlation*”[tiab] OR error[tiab] OR errors[tiab] OR “individual variability”[tiab] OR “interval variability”[tiab] OR “rate variability”[tiab] OR (variability[tiab] AND (analysis[tiab] OR values[tiab])) OR (uncertainty[tiab] AND (measurement[tiab] OR measuring[tiab])) OR “standard error of measurement”[tiab] OR sensitiv*[tiab] OR responsive*[tiab] OR (limit[tiab] AND detection[tiab]) OR “minimal detectable concentration”[tiab] OR interpretab*[tiab] OR ((minimal[tiab] OR minimally[tiab] OR clinical[tiab] OR clinically[tiab]) AND (important[tiab] OR significant[tiab] OR detectable[tiab]) AND (change[tiab] OR difference[tiab])) OR (small*[tiab] AND (real[tiab] OR detectable[tiab]) AND (change[tiab] OR difference[tiab])) OR “meaningful change”[tiab] OR “ceiling effect”[tiab] OR “floor effect”[tiab] OR “Item response model”[tiab] OR IRT[tiab] OR Rasch[tiab] OR “Differential item functioning”[tiab] OR DIF[tiab] OR “computer adaptive testing”[tiab] OR “item bank”[tiab] OR “cross-cultural equivalence”[tiab]).

(#1 AND #2 AND #3) NOT (‘delphi-technique’[ti] OR cross-sectional[ti] OR “addresses”[Publication Type] OR “biography”[Publication Type] OR “case reports”[Publication Type] OR “comment”[Publication Type] OR “directory”[Publication Type] OR “editorial”[Publication Type] OR “festschrift”[Publication Type] OR “interview”[Publication Type] OR “lectures”[Publication Type] OR “legal cases”[Publication Type] OR “legislation”[Publication Type] OR “letter”[Publication Type] OR “news”[Publication Type] OR “newspaper article”[Publication Type] OR “patient education handout”[Publication Type] OR “popular works”[Publication Type] OR “congresses”[Publication Type] OR “consensus development conference”[Publication Type] OR “consensus development conference, nih”[Publication Type] OR “practice guideline”[Publication Type]) NOT (“animals”[MeSH Terms] NOT “humans”[MeSH Terms]).

Filters: Publication date from 2013/07/01.

### Embase search June 19, 2014

#1 instruments:ti,ab OR scales:ti,ab OR questionnaires:ti,ab OR measures:ti OR methods:ti OR outcome-measurements:ti,ab OR (tests:ti,ab AND review:ti,ab) OR ‘outcomes research’/de OR ‘treatment outcome’/de OR ‘psychologic test’/de OR ‘measurement’/de OR ‘functional assessment’/de OR ‘pain assessment’/de OR ‘questionnaire’/de OR ‘rating scale’/de.

#2 review:ti OR (literature AND search*) OR (medline AND search*) OR ‘systematic review’/exp.

#3 ‘intermethod comparison’/exp OR ‘data collection method’/exp OR ‘validation study’/exp OR ‘feasibility study’/exp OR ‘pilot study’/exp OR ‘psychometry’/exp OR ‘reproducibility’/exp OR reproducib*:ab,ti OR ‘audit’:ab,ti OR psychometr*:ab,ti OR clinimetr*:ab,ti OR clinometr*:ab,ti OR ‘observer variation’/exp OR ‘observer variation’:ab,ti OR ‘discriminant analysis’/exp OR ‘validity’/exp OR reliab*:ab,ti OR valid*:ab,ti OR ‘coefficient’:ab,ti OR ‘internal consistency’:ab,ti OR (cronbach*:ab,ti AND (‘alpha’:ab,ti OR ‘alphas’:ab,ti)) OR ‘item correlation’:ab,ti OR ‘item correlations’:ab,ti OR ‘item selection’:ab,ti OR ‘item selections’:ab,ti OR ‘item reduction’:ab,ti OR ‘item reductions’:ab,ti OR ‘agreement’:ab,ti OR ‘precision’:ab,ti OR ‘imprecision’:ab,ti OR ‘precise values’:ab,ti OR ‘test–retest’:ab,ti OR (‘test’:ab,ti AND ‘retest’:ab,ti) OR (reliab*:ab,ti AND (‘test’:ab,ti OR ‘retest’:ab,ti)) OR ‘stability’:ab,ti OR ‘interrater’:ab,ti OR ‘inter-rater’:ab,ti OR ‘intrarater’:ab,ti OR ‘intra-rater’:ab,ti OR ‘intertester’:ab,ti OR ‘inter-tester’:ab,ti OR ‘intratester’:ab,ti OR ‘intra-tester’:ab,ti OR ‘interobeserver’:ab,ti OR ‘inter-observer’:ab,ti OR ‘intraobserver’:ab,ti OR ‘intra-observer’:ab,ti OR ‘intertechnician’:ab,ti OR ‘inter-technician’:ab,ti OR ‘intratechnician’:ab,ti OR ‘intra-technician’:ab,ti OR ‘interexaminer’:ab,ti OR ‘inter-examiner’:ab,ti OR ‘intraexaminer’:ab,ti OR ‘intra-examiner’:ab,ti OR ‘interassay’:ab,ti OR ‘inter-assay’:ab,ti OR ‘intraassay’:ab,ti OR ‘intra-assay’:ab,ti OR ‘interindividual’:ab,ti OR ‘inter-individual’:ab,ti OR ‘intraindividual’:ab,ti OR ‘intra-individual’:ab,ti OR ‘interparticipant’:ab,ti OR ‘inter-participant’:ab,ti OR ‘intraparticipant’:ab,ti OR ‘intra-participant’:ab,ti OR ‘kappa’:ab,ti OR ‘kappas’:ab,ti OR ‘coefficient of variation’:ab,ti OR repeatab*:ab,ti OR (replicab*:ab,ti OR ‘repeated’:ab,ti AND (‘measure’:ab,ti OR ‘measures’:ab,ti OR ‘findings’:ab,ti OR ‘result’:ab,ti OR ‘results’:ab,ti OR ‘test’:ab,ti OR ‘tests’:ab,ti)) OR generaliza*:ab,ti OR generalisa*:ab,ti OR ‘concordance’:ab,ti OR (‘intraclass’:ab,ti AND correlation*:ab,ti) OR ‘discriminative’:ab,ti OR ‘known group’:ab,ti OR ‘factor analysis’:ab,ti OR ‘factor analyses’:ab,ti OR ‘factor structure’:ab,ti OR ‘factor structures’:ab,ti OR ‘dimensionality’:ab,ti OR subscale*:ab,ti OR ‘multitrait scaling analysis’:ab,ti OR ‘multitrait scaling analyses’:ab,ti OR ‘item discriminant’:ab,ti OR ‘interscale correlation’:ab,ti OR ‘interscale correlations’:ab,ti OR (‘error’:ab,ti OR ‘errors’:ab,ti AND (measure*:ab,ti OR correlat*:ab,ti OR evaluat*:ab,ti OR ‘accuracy’:ab,ti OR ‘accurate’:ab,ti OR ‘precision’:ab,ti OR ‘mean’:ab,ti)) OR ‘individual variability’:ab,ti OR ‘interval variability’:ab,ti OR ‘rate variability’:ab,ti OR ‘variability analysis’:ab,ti OR (‘uncertainty’:ab,ti AND (‘measurement’:ab,ti OR ‘measuring’:ab,ti)) OR ‘standard error of measurement’:ab,ti OR sensitiv*:ab,ti OR responsive*:ab,ti OR (‘limit’:ab,ti AND ‘detection’:ab,ti) OR ‘minimal detectable concentration’:ab,ti OR interpretab*:ab,ti OR (small*:ab,ti AND (‘real’:ab,ti OR ‘detectable’:ab,ti) AND (‘change’:ab,ti OR ‘difference’:ab,ti)) OR ‘meaningful change’:ab,ti OR ‘minimal important change’:ab,ti OR ‘minimal important difference’:ab,ti OR ‘minimally important change’:ab,ti OR ‘minimally important difference’:ab,ti OR ‘minimal detectable change’:ab,ti OR ‘minimal detectable difference’:ab,ti OR ‘minimally detectable change’:ab,ti OR ‘minimally detectable difference’:ab,ti OR ‘minimal real change’:ab,ti OR ‘minimal real difference’:ab,ti OR ‘minimally real change’:ab,ti OR ‘minimally real difference’:ab,ti OR ‘ceiling effect’:ab,ti OR ‘floor effect’:ab,ti OR ‘item response model’:ab,ti OR ‘irt’:ab,ti OR ‘rasch’:ab,ti OR ‘differential item functioning’:ab,ti OR ‘dif’:ab,ti OR ‘computer adaptive testing’:ab,ti OR ‘item bank’:ab,ti OR ‘cross-cultural equivalence’:ab,ti.

(#1 AND #2 AND #3) NOT ‘Delphi technique’:ti OR Cross-sectional:ti OR ‘case report’/de OR letter:it OR animal/exp OR ‘animal model’/exp OR ‘animal experiment’/exp.

Filters: Publication date from 2013/07/01.
